# High-throughput thermal denaturation of tryptophanyl-tRNA synthetase combinatorial mutants reveals high-order energetic coupling determinants of conformational stability

**DOI:** 10.1063/4.0000182

**Published:** 2023-08-23

**Authors:** Violetta Weinreb, Gabriel Weinreb, Charles W. Carter

**Affiliations:** Department of Biochemistry and Biophysics, University of North Carolina at Chapel Hill, Chapel Hill, North Carolina 27599-7260, USA

## Abstract

Landscape descriptions provide a framework for identifying functionally significant dynamic linkages in proteins but cannot supply details. Rate measurements of combinatorial mutations can implicate dynamic linkages in catalysis. A major difficulty is filtering dynamic linkages from the vastly more numerous static interactions that stabilize domain folding. The *Geobacillus stearothermophilus* (TrpRS) D1 switch is such a dynamic packing motif; it links domain movement to catalysis and specificity. We describe Thermofluor and far UV circular dichroism melting curves for all 16 D1 switch variants to determine their higher-order impact on unliganded TrpRS stability. A prominent transition at intermediate temperatures in TrpRS thermal denaturation is molten globule formation. Combinatorial analysis of thermal melting transcends the protein landscape in four significant respects: (i) bioinformatic methods identify dynamic linkages from coordinates of multiple conformational states. (ii) Relative mutant melting temperatures, δT_M_, are proportional to free energy changes. (iii) Structural analysis of thermal melting implicates unexpected coupling between the D1 switch packing and regions of high local frustration. Those segments develop molten globular characteristics at the point of greatest complementarity to the chemical transition state and are the first TrpRS structures to melt. (iv) Residue F37 stabilizes both native and molten globular states; its higher-order interactions modify the relative intrinsic impacts of mutations to other D1 switch residues from those estimated for single point mutants. The D1 switch is a central component of an escapement mechanism essential to free energy transduction. These conclusions begin to relate the escapement mechanism to differential TrpRS conformational stabilities.

## I. INTRODUCTION

Differential conformational stability strongly influences the TrpRS catalytic cycle.^1–4^ Catalytic assist by Mg^2+^ ion, on the one hand, depends entirely on its coupling to domain motions,^3,5,6^ hence on the conformational free energy profile. On the other hand, the free energy change for the catalytic conformational change is positive in the presence of the bound pyrophosphate product, PPi, opposing it unless PPi has dissociated.^7^ Coupling assembly of the fully active catalytic apparatus tightly to domain motion, while ensuring that domain motion is unfavorable without PPi release, couples conformational changes reciprocally to the chemistry of ATP utilization. We have described this reciprocally coupled gating behavior as an “escapement mechanism”^3,4^ by analogy to the mechanism allowing the verge and foliot in a mechanical clock to be driven in discrete steps of equal time intervals by the crown gear. It enhances the efficiency of vectorial ATP utilization^8,9^ and is, thus, a potentially general model for energetic coupling in a broader range of NTP-dependent transducing enzymes.^10,11^ That deepened understanding sheds light on how biological processes are self-sustaining far from equilibrium.^4,12,13^

**FIG. 1.**
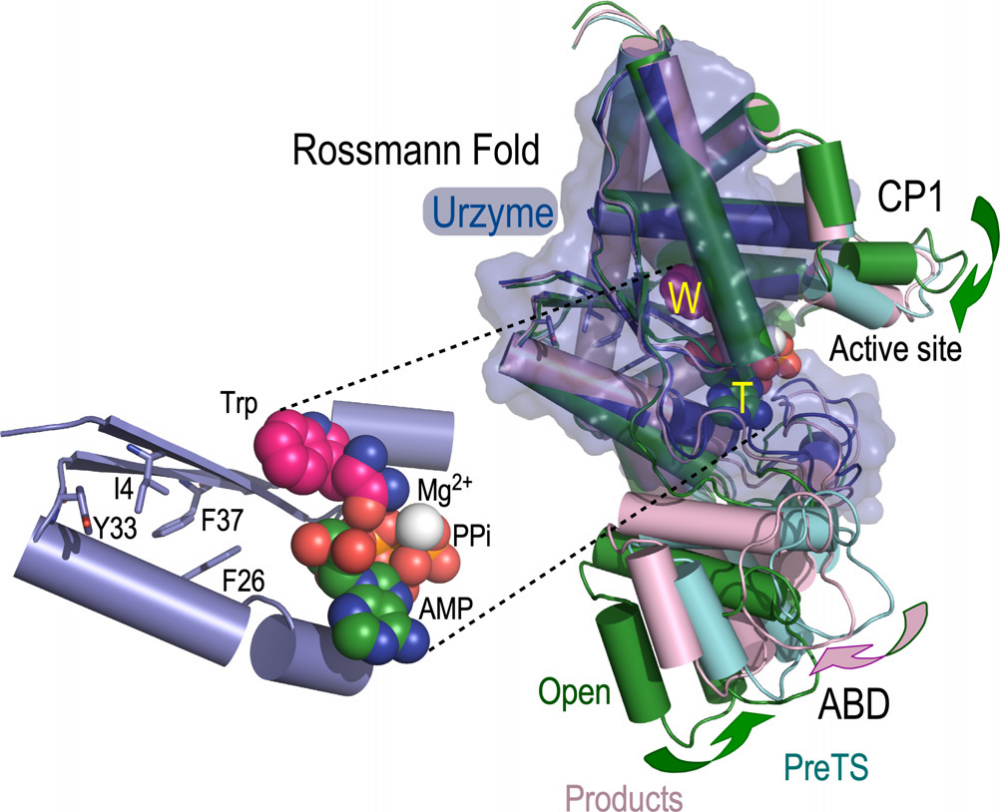
The D1 switch mediates relative movements of two rigid bodies.^51^ Multiple crystal structures show that the anticodon-binding domain (ABD) and Connecting Peptide 1 (CP1) move essentially as rigid bodies relative to the urzyme (blue) throughout the catalytic cycle. T and W indicate substrates Mg^2+^·ATP and tryptophan, respectively, in the active site. Substituting tryptophanamide for tryptophan locks TrpRS into a Pre-transition-state (PreTS) conformation (PDB ID 1MAU; colored cyan here). Conformational changes: (i) active site assembly from the open state (Forest) to the high-energy PreTS state entails hinge-bending closure plus twisting of the CP1 and ABD domains (solid curved green arrows); (ii) the catalytic step involves an untwisting motion of the ABD (magenta solid curved arrow). The D1 switch (enlargement) mediates shear during these conformational changes. The four D1 switch residues repack during both transitions.

Experimental and computational data supporting this escapement mechanism were derived from strategically selected combinatorial mutations of residues in the D1 switch (Fig. 1), a broadly conserved^14^ tertiary packing motif in the first β-α-β crossover connection of most Rossmannoid proteins. It mediates shear developed during TrpRS domain motion.

To study how interactions between these residues impact catalysis, variants of four D1 switch residues—I4V, F26L, Y33F, and F37I—were designed using Rosetta^3,15^ to minimize stability differences between the excited Pre-transition (PreTS) conformational state stabilized by trytophanamide and ATP and the lower-energy preceding and subsequent ground-state structures. When assayed in the presence of Mg^2+^ and Mn^2+^, wild type TrpRS and the 15 combinatorial TrpRS mutants represent a full, 2^5^ factorial design, including all possible perturbations of the four variants suggested by Rosetta and their coupling to the catalytic metal ion.

Multidimensional thermodynamic cycle analysis of both steady state^3,16^ and single turnover^1^ kinetics of tryptophan activation revealed that the five-way energetic coupling of all four D1 residues to the active-site Mg^2+^ ion contributes −5.5 kcal/mole to transition-state stabilization during tryptophan activation^3^ and ∼−5.0 kcal/mole to the relative rates of tryptophan vs tyrosine activation.^16^ The resulting allosteric behavior, therefore, accounts for both high catalytic proficiency and the requisite specificity of tryptophan activation.

These highly cooperative, long-range catalytic effects suggest that dynamic repacking in the D1 switch^1,17^ senses and communicates the domain configuration to the active-site Mg^2+^ ion, switching it to a catalytically active position only as the domains untwist. We validated that conclusion by showing that a modular thermodynamic cycle constructed from the full-length TrpRS, TrpRS urzyme, and intermediate constructs made by restoring each of the two domains deleted in the urzyme yields the same coupling energy— ∼−5 kcal/mole—between the two domains.^18^ Mg^2+^-assisted catalysis, thus, arises entirely from domain motions driven by differential conformational stability changes induced by the succession of bound ligands, as the chemical events proceed.^2,3,16^

We previously^3^ measured melting curves only for the four single point mutants, which precluded estimating higher-order coupling energies. The ensemble of variant TrpRS proteins provides structural perturbations to probe in unprecedented detail the, as yet unknown, coupling between differential conformational stability and catalysis. High-throughput thermal melting measurements for the ensemble over the conformational range stabilized by different ligands present a paradigm for high-resolution mapping of functionally relevant structure–stability relationships.

Microcalorimetric measurements of thermal melting reported in the accompanying paper^19^ showed that *Geobacillus stearothermophilus* tryptophanyl-tRNA synthetase (TrpRS) melts over broad temperature ranges (25 °C in the absence of ligands; 15 °C when bound to the inhibitory complex with tryptophanamide and ATP). Three successive transitions have substantial heat capacity changes. This work extends those observations to higher throughput stability assays using Thermofluor^20,21^ and far UV circular dichroism (CD) in order to characterize the melting of the 16 combinatorial mutational variants. Evidence on how the four side chains cooperate to produce catalysis requires comparing native and all 15 variants (see supplement to Ref. 3). Although these stability measures probe distinct physical changes from those detected by microcalorimetry, they exhibit qualitatively similar behavior. Moreover, regression methods allow us to evaluate intrinsic and all higher-order—six two-way, four three-way, and one four-way—interactions that influence stability. We describe four main results:

(i) The new probes confirm that all TrpRS variants denature via a molten globular intermediate—defined both by enhanced fluorescence of the Sypro Orange dye in Thermofluor scans and by the quantitative increase in R_g_ from simulations. The molten globule forms at temperatures ∼8° lower than those required to melt secondary structures.
(ii) The multi-step experimental melting behavior matches that from a computational free energy surface of a TrpRS monomer liganded to form the PreTS state.^19^ That trajectory is reversible by definition because the monomer at infinite dilution cannot aggregate. Simulations also furnish valuable structural metrics and cameo snapshots that aid interpretation of melting behavior.
(iii) Mutation induces temperature perturbations that agree closely with those computed from linear regression models based on the design matrix of mutated sites. The near zero residual implies that variant stabilities form a thermodynamic cycle using a formula described by Calvin *et al.*^22^ relating perturbations in T_m_ and δT_m_ to perturbations in folding free energy, δΔG.
(iv) Wild type residue F37 from the D1 switch stabilizes both native and molten globular states. Its higher-order interactions modify the relative intrinsic impacts of mutations to other D1 switch residues from those estimated for single point mutants.

We hope to extend effects reported here for unliganded TrpRS by examining how the mutant design matrix correlates relative stabilities of different conformations encountered along the catalytic structural profile with steady-state and pre-steady state kinetic properties already measured,^1,3^ hence to identify their highly cooperative contribution to the escapement mechanism.

## II. METHODS

### A. Expression and purification

His_6_-N-terminally tagged and untagged WT TrpRS and mutant proteins were expressed in *E. coli* BL21(DE3)pLysS, with kanamycin and chloramphenicol. Cells were resuspended in 20 mM HEPES pH 7.6, 0.3 M NaCl_2_, 10 mM BME, and 30 mM imidazole (2.5 ml/g wet weight). Cell pastes were sonicated and cleared by centrifugation at 15 000 rpm for 30 min at 4 °C. The cleared lysate was mixed with Ni beads (Thermo Scientific HisPur™ Ni-NTA Resin) at approximately 2–3 ml per liter of lysate, and the suspension passed into a self-flowing glass column. After washing with 20 column volumes, His-tagged protein was eluted with 20 mM HEPES pH 7.6, 0.3 M NaCl_2_, 10 mM BME, and 0.3 M imidazole. Imidazole concentrations were reduced by dilution to 30 mM before incubating overnight with TEV protease at a ratio of 1:10. Purified, cleaved proteins were concentrated using an Amicon PM10 Ultra membrane and stored at −20° C in 50% glycerol. We performed roughly 30% of the experiments on both tagged and untagged proteins, without significant differences. We report here only results using cleaved proteins.

### B. Combinatorial mutagenesis

D1 switch mutations were derived by a process that that has been described previously.^2,15^ Because the method is quite general and has proven so useful, we describe it again in outline. Delaunay tessellation of the set of side chain centroids in any crystal structure provides a unique decomposition of side chain packing into simplices of four nearest-neighbors. We tessellated TrpRS crystal structures 12DR (Open unliganded 6 monomers/a.u.), 1MAW (OPEN, ATP complex, 6 monomers/a.u.), 1MB2 (Open, tryptophan complex, 6 monomers/a.u.), 1MAU (PreTS 1 monomer/a.u.), and 1I6K, 1I6L, and 1I6M (Products complexes each with 1 monomer/a.u.) and accumulated likelihood scores as described.^23,24^ The crystallographically distinct tessellations of the 18 open structures agreed on >96% of the simplices, establishing the robustness of the technique. A sorting algorithm (DIFF, Stephen Cammer, unpublished) then identified those simplices that differed in the three conformational states. These dozen or so “dynamic” Delaunay simplices occurred in ∼4 clusters distributed throughout the TrpRS monomer which we named Dn, where n = 1–4 was inversely related to the numbers of residues involved in each cluster. The D1 cluster had seven residues. Cammer^14^ independently identified the D1 cluster as one of the most prominent side chain packing motifs in the proteome, as it occurs in the first crossover connection of Rossmannoid folds.

A complementary study used Rosetta^25^ to identify mutations expected to decrease the high conformational energy of PreTS TrpRS,^26^ relative to the Open ground^27^ and Product^28,29^ states identified by x-ray crystallography. That study^15^ produced a second list of conformationally sensitive side chains independently of the Delaunay tessellation pattern. Remarkably, the two sets of dynamic residues included many of the same residues. Moreover, the Rosetta multi-state design analysis obviated the need to perform scanning alanine mutagenes by suggesting specific mutations for each site, whose physical properties would change the relative stabilities of the various TrpRS conformations encountered along the reaction profile. Selected mutations to four of seven non-polar residues in the D1 switch,^15^ I4V, F26L, Y33F, and F37I, became extraordinarily useful in subsequent combinatorial mutagenesis.^1–3,16,18,30^

### C. Thermofluor stability assays

Differential scanning fluorimetry (Thermofluor) was developed as a high-throughput method to compare ligand affinity from the elevation of melting temperatures.^20,21,31^ Thermofluor is especially useful for our purposes because it is performed rapidly in 384-well microtiter plates. A 6:5000 dilution of Sypro Orange dye into 20 mM HEPES, 50 mM NaCl_2_ (pH 7.0) buffer was mixed with 20 *μ*l of a 6 *μ*Mol enzyme solution, with 20 mM MgCl_2_. No ligands were added. Each sample was replicated six times on the 384-well format plate, which was heated from 25 to 95 °C in 0.4° steps lasting 8.7 s. We made fluorescence measurements with an ABI 7900HTFast Real-Time PCR (Polymerase Chain Reaction) instrument, and analyzed those data either with SigmaPlot or with MATLAB programs described further below. One-off melting curves using ANS (1-Anilino-8-naphthalene sulfonate) were performed using a SPEX Fluorolog-3 spectrofluorometer. Identical sixfold replicated measurements were performed with mutant proteins purified on two different occasions, substantially reducing the overall variance of estimated melting temperatures. Subsequent experiments were also performed in this manner with 1 mM MgCl_2_, 1 mM MnCl_2_, and without added metals (see Sec. 7 of Ref. 53).

### D. Circular dichroism (CD)

Circular dichroism measurements were done on an Applied Photophysics Chirascan Plus steady-state Circular Dichroism instrument in the UNC Macinfac facility. We measured the ellipticity of 8 *μ*M of each protein in 200 mM Sodium Phosphate Buffer (pH 7.3) without added metals from 25 to 90 °C at 221 nm (θ_221_) and similarly at 270 nm (θ_270_).

### E. Data reduction and parameter estimation

Both Thermofluor and CD melting curves were processed using Matlab^®^ (Mathworks).^32^ One of us, GW, built the software as a pipeline of .m-files that reduce and analyze the data, including thermodynamic characterization and presentation of statistics. The pipeline consists of three parts:

1. Reading data from high-throughput RT PCR files and transforming them into a matrix with four columns: (i) well number; (ii) an index representing the mutation [we assigned all mutants a number so the wild type was indexed by 0, I4V by 1, and so on, according to the sequence number of the mutation(s)]; and, finally, the data, (iii) temperature and (iv) fluorescence reading.
2. Fitting the data (both Thermofluor and CD).
3. Estimation of $T_{m}$ by the “model” and “ratio” methods (see Sec. S4 of Ref. 53).

The software determines temperatures at the low and high levels of the sigmoidal part of the experimental curve. If these cannot be found, the user is prompted to input them manually. These positions determine the intervals for the initial and final linear baselines and the sigmoidal segment [Eqs. (S1) and (S4) of Ref. 53]. These intervals then are used for the initial parameter estimation in the “Model” scenario and to extrapolate the initial and final parts to utilize the “Ratio” method. In practice, there was very little difference between parameters estimated by the two methods. An additional module estimates T_m_ using a thermodynamic module. The program allows for repeated analysis and/or re-initialization for problematic datasets if needed. Matlab codes are available at https://github.com/cwcarter/Thermafuor-data-reduction.^33^

Outputs are error log, parameter file, an output Excel data file with fitting and simulation parameters, and an Excel file where replicates are averaged and recorded with standard deviations and errors.

### F. Assessing thermodynamic significance of melting temperatures in the face of aggregation

Aggregation complicates thermodynamic interpretations of denaturation experiments. A requisite for attributing thermodynamic significance to denaturation experiments has always been that melting curves be reversible. Many proteins, however, fail to meet this exacting criterion because their unfolded configurations aggregate to varying degrees at the concentrations necessary for high signal-to-noise measurements. TrpRS exhibits this frustrating behavior (Sec. 1 of Ref. 53). We argue here that the underlying processes are largely reversible, and that errors induced in melting temperature by aggregation are small, relative to the significant signal in melting temperatures:

(i) Controls described in Sec. 1 of Ref. 53 and illustrated Fig. S1 show that the time interval between temperature changes should allow sufficient time for equilibration at each temperature [Fig. S1(b)], and document recovery of some TrpRS activity, despite partial losses owing to aggregation of denatured TrpRS [Figs. S1(c) and S1(d)].
(ii) Regression models (Section J) relating melting temperatures to the mutational design matrix suggest that the error induced by aggregation is <5%.

### G. Protein concentration effects

TrpRS is a functional dimer that introduces another complicating factor because melting curves will depend on some extent on protein concentrations related to the dimer dissociation constant, which must also interact with monomer denaturation. The dimer dissociation constant measured directly from monomer/dimer distributions using atomic force microscopy is K_D_ = 5 nM in a similar buffer.^34^ As we measure melting at much higher concentrations (i.e., 6–8 *μ*M), we expect this effect to be minimal as well. Section 2 of Ref. 53 discusses experimental evidence that *T_m_,*s measured over a fourfold concentration range differs by <1 °C (Fig. S2).

### H. Free energy calculations

Calvin *et al.*^22^ derived a linear relationship between the proportional change in melting temperature and the incremental free energy change induced by perturbing a reversible equilibrium between two differently folded states of a macromolecule [Eq. (1)],

$$\delta T_{m}/T_{m0}=\;\delta G/\Delta H,$$ (1)

where T_m0_ is the melting temperature of a reference, or WT TrpRS, and ΔH is the overall enthalpy change of its structural transition. The total enthalpy change ΔH_tot_ = −46 kcal/mole for complete unfolding.^19^ As enthalpy is a state function, ΔH_tot_ is the sum of the enthalpy changes, ΔH_φ_ and ΔH_θ_, of forming the molten globule and melting the helices. Voight integration of individual peaks in the experimental melting curves (see Fig. 1 of Ref. 19) provides estimates of the proportion of the total overall ΔH of melting for each of the three transitions. Those estimates (see Table SIII of Ref. 53) are ΔH_φ_ = −27 kcal/mole for molten globule formation and ΔH_θ_ = −11 kcal/mole for helix melting. Free energies in Table I are based on this conversion.

**TABLE I.** Regression coefficients and Student test P values of predictive models illustrated in Fig. 4 for ${T_{m}}_{\varphi }$ and ${T_{m}}_{\theta }$. R^2^ values for the models are 0.97 and 0.94, and root mean squared errors are 0.31° and 0.39°. Coefficients with 0.05 < P < 1.0 were estimated by default because higher-order terms are more significant; those with P > 0.5 are listed as not significant (ns). Δ(ΔGφ, θ) were computed using Eq. (1).

Predictor	Δ(ΔGφ) (kcal/mole)	Pφ	Δ(ΔGθ) (kcal/mole)	Pθ
I4	0.08	0.0928	0.0006	0.9607
F26	0.04	0.393	0.09	<0.0001^*^
Y33	0.04	0.3632	−0.05	0.0006^*^
F37	−1.05	<0.0001^*^	−0.32	<0.0001^*^
I4^*^F26	−0.09	0.3215	ns	
I4^*^Y33	0.09	0.335	0.00	0.6585
I4^*^F37	ns		ns	
F26^*^Y33	0.48	<0.0001^*^	−0.11	0.0004^*^
F26^*^F37	ns		−0.004	0.8826
Y33^*^F37	ns		−0.02	0.5244
I4^*^F26^*^Y33	0.66	0.0011^*^	ns	
I4^*^F26^*^F37	ns		ns	
I4^*^Y33^*^F37	ns		0.13	0.0198^*^
F26^*^Y33^*^F37	ns		−0.15	0.0083^*^
I4^*^F26^*^Y33^*^F37	ns		ns	

### I. Regression modeling^3,5^

Horovitz and Fersht outlined how multiple mutant cycles give evidence for energetic coupling between sites by evaluating all intersecting two-way thermodynamic cycles.^35^ Misconceptions, nonetheless, persist arising from confusion between the actual experimental observations for individual multiple mutants themselves and the coupling energies they imply. Energetic coupling between residues depends on how the effect of a particular mutation changes in different contexts (wild type, mutations at other sites, etc.) in which it is observed. For that reason, the entire set of combinatorial mutants must be analyzed as an ensemble. Comparison between a quadruple mutant and WT protein may suggest only a small interaction when, in fact, the overall coupling can be very significant. Our own work^3,5^ and that of others^36^ provide examples of how multi-mutant thermodynamic cycles can be used to derive higher-order coupling energies.

The mutational design matrix implies a set of simultaneous linear equations for *T_mcalc,i_*,

$$T_{m\;\;\text{calc},i}=\;\text{Constant}\;+\;\Sigma_{\text{ijkl}}\left(\beta_{i}*F_{i}+\;\beta_{\text{ij}}*F_{i}*F_{j}+\;\beta_{\text{ijk}}*F_{i}*F_{j}*F_{k}+\;\beta_{\text{ijkl}}*F_{i}*F_{j}*F_{k}*F_{l}\right)+\varepsilon ,$$ (2)

where βs are coefficients in degrees,^37^ ijkl = {0 or 1} are binary elements of the design matrix describing the presence or absence of each mutation (see the barcode in Fig. 5), and **ε** = { $T_{m\;\mathrm{calc}}-T_{m\;\mathrm{obs}}\}$ is a difference vector. Regression modeling to solve the implicit simultaneous equations (2) greatly reduces the computational tedium of working out individual two-way thermodynamic cycles. Adjusting coefficients to minimize the total squared residual is straightforward in many statistics programs, e.g., JMP,^38^ and leads simultaneously to maximum-likelihood estimates of the coefficients, their standard errors, and P-values. Such calculations are otherwise equivalent to estimating coupling effects individually, as described.^35^

## III. RESULTS

### A. TrpRS denatures via an intermediate, molten globular state

**FIG. 2.**
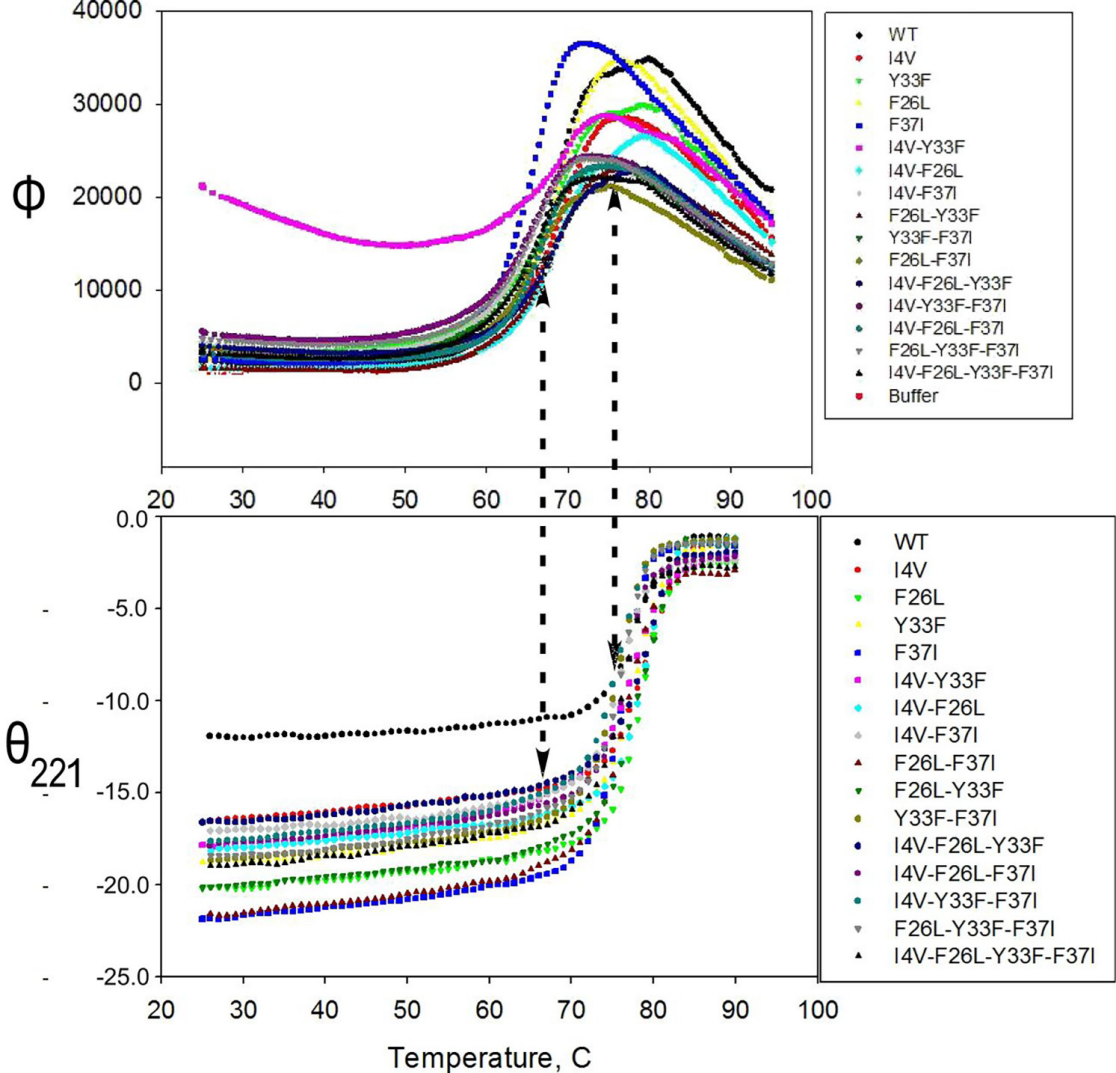
Raw data for all thermal melting processes. Vertical dashed arrows align the midpoints of the two transitions with corresponding progress along the other process. Note that the melting of α-helices does not begin until the midpoint of the molten globular transition.

All unliganded variants observed by Thermofluor and CD at 221 nm (θ_221_) exhibit successive, linked, quasi-two-state melting processes leading ultimately to the loss of secondary structure (Fig. 2). $T_{m}$ estimates from fitting to a conventional two-state model^39^ are compared in Table SI of Ref. 53 with those estimated by the ratio method (Fig. S3). Variances from fitting the CD melting curves are comparable to those obtained by averaging sixfold replication in the Thermofluor 384-well plates and from repetition of those plates on two different dates (February and May) with different TrpRS preparations. The mean increase for all mutants between $T_{m_{\Theta }}$ vs $T_{m\Phi }$ is 8.5 ± 0.8 °C. As noted later, the range of values, 0.8 °C, arises mostly from significant differences in the behaviors of individual variants, rather than from experimental errors. Mg^2+^ ion lowers the Thermofluor melting temperature of all variants by ∼2.5 °C. The effects of divalent metal ions on Thermofluor melting curves are discussed further in Sec. S7 of Ref. 53 and illustrated in Fig. S5. Ellipticity at 221 nm arises from α-helical secondary structures, whereas Thermofluor detects partial breakdown of native-like non-polar side chain packing, which allows penetration of dye into the core. The latter process is related to the loss of ellipticity at 270 nm [Fig. S1(c)], which arises from persistent asymmetries in the relative positions of aromatic sidechains.^40^

### B. Microcalorimetry, thermofluor, and θ_221_ detect physically different changes

**FIG. 3.**
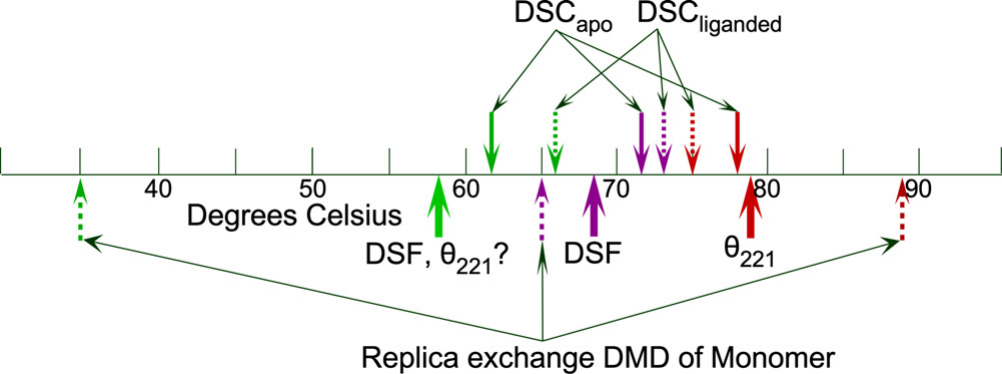
Melting phenomena detected by different stability probes. The unusually wide range observed in the simulated melting of the monomer (bottom thin dashed lines) has been attributed to the lack of coordination present in the TrpRS dimer.^19^ The bold arrows below the thermal scale represent the mean behavior detected for the ensemble of TrpRS variants. The transition about which this work reveals the most is the middle transition to a molten globule, detected by DSF and shown in purple. The question mark associated with the lowest temperature transition detected by DSF and far UV CD signifies that this transition is apparent only when considering all variants as an ensemble.

Significant TrpRS melting occurs over a wide temperature range. Nevertheless, each detection method exhibits a similar pattern of three successive transitions, as were observed in simulated melting^19^ (Fig. 3).

### C. All measurements are consistent with three-state unfolding

State probabilities can be estimated as a function of temperature as described in Sec. 6 of Ref. 53 (Fig. S4). Separation of two melting processes implies that the second melting process requires prior loss of native-like core packing, according to a three-state scheme. Cremades and Sancho describe similar results with flavodoxin,^41^ a related Rossmannoid protein. Melting transitions detected by Thermofluor and θ_221_ (Fig. 2) show that α-helices do not begin to melt until the molten globule concentration reaches a maximum and melt completely only when the molten globule concentration is exhausted. Ptitsyn and co-workers^40^ reported a similar observation for α-lactoglobulin. All mutants show a pattern (Fig. S4 of Ref. 53) in which (a) P_2max_ is close to 1.0, and (b) the probabilities P_1_ and P_3_ at P_2max_ are close to 0.

Two different probes identify transitions between states at two different temperatures. They, therefore, rule out two-state denaturation and strongly imply formation of an intermediate, molten globular state.^42^ TrpRS thermal denaturation, therefore, appears to be a three-state process involving two successive transitions, each of which is two-state to good approximation, and for which the intermediate molten globule state is the reactant in the second.

Stabilization or (less likely) destabilization of the molten globular state by dye binding would change the concentration of the reactant for the final unfolding of the helices in a regime where those changes occur most rapidly—i.e., where sypro orange fluorescence is changing most rapidly with temperature. Any change in $T_{m\;}\varphi$, in turn, would affect the concentration of molecules that give rise to the final unfolding process and be expected to alter the earliest part of the CD melting curve. The fact that the CD melting curves with and without sypro orange cannot be distinguished over any of their sigmoidal shape, thus, argues that the dye has no detectable influence on the formation of the molten globular state [Fig. S1(e) of Ref. 53]. Thus, although Sypro Orange may stabilize the molten globule very weakly, the effect is small enough to ignore for our purposes.

**FIG. 4.**
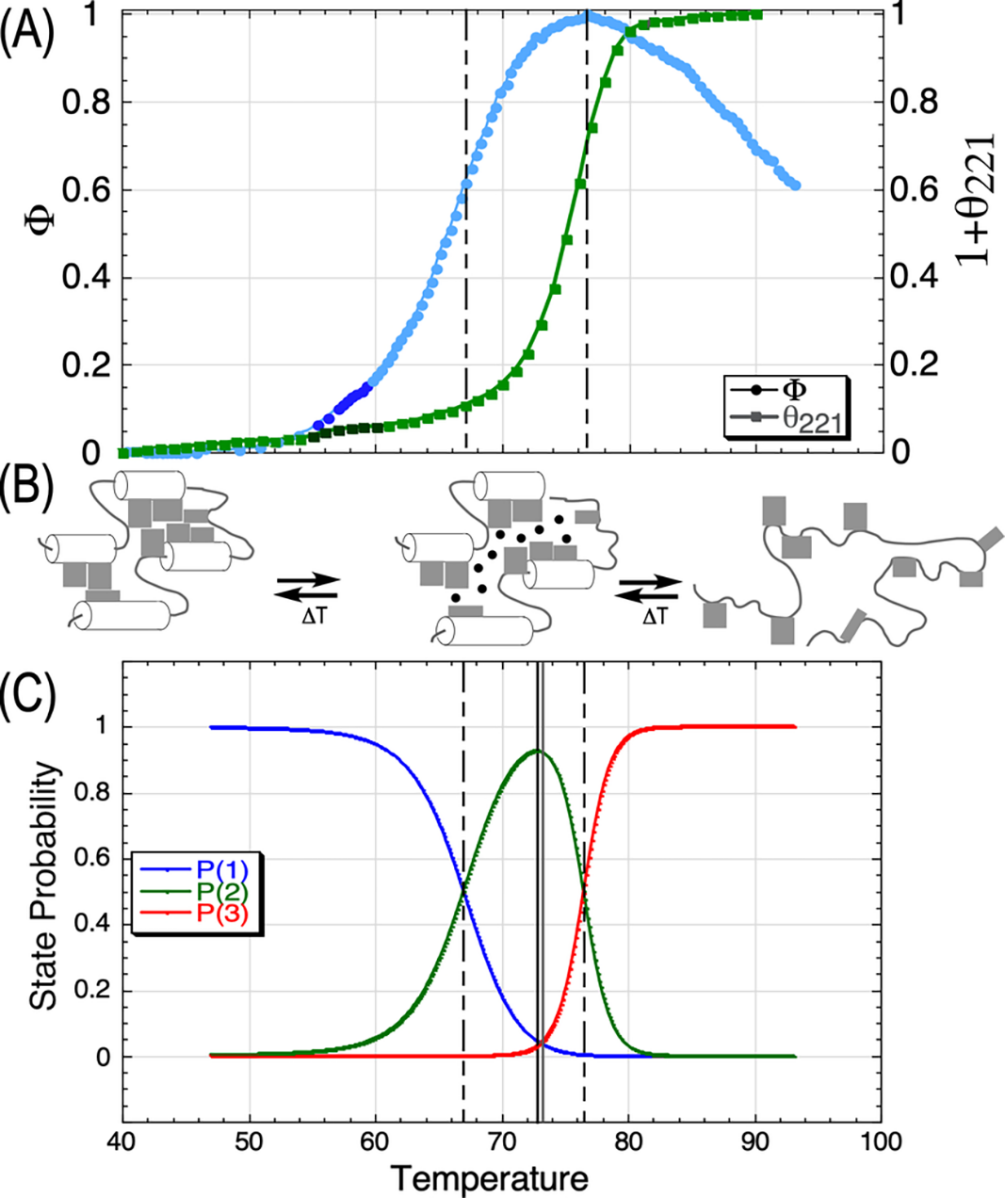
Quantitative metrics supporting a three-state model for native TrpRS denaturation. (a) Normalized experimental Thermofluor (circles) and CD (squares) melting curves. The same temperature scale in degrees Celsius applies to all three panels. Bright-colored data points between 56 and 60 °C suggest detection of the broad low temperature transition seen in experimental and computational heat capacity curves.^19^ (b) Dominant structural ensembles below $T_{m\varphi }$ (Thermofluor) resemble the native state, S1. Between $T_{m\varphi }$ and $T_{m\theta }$ (CD), they resemble molten globule, S2, and above $T_{m\theta },$ they are characterized by loss of α-helices. Vertical lines represent melting points detected by the two datasets. (c) Probabilities (P_1_, P_2_, and P_3_) of the three states represented in B (Sec. 4 of Ref. 53). The temperatures and amplitudes of P2 and the intersection of P_1_ = P_3_ are unconstrained, hence define quantitative metrics of the validity of the three-state approximation.

The three-state model ignores for the moment an initial transition seen in heat capacity profiles. Moreover, the dark blue data points in the DSF (Differential Scanning Fluorimetry) and far UV CD melting curves [Fig. 4(a)] both exhibit a reproducible signal that may also represent that transition. The impact of that transition on the two probe metrics is too modest to identify with confidence in any single scan. However, when converted to first derivative and analyzed as an ensemble, the DSC (Differential Scanning Calorimetry) melting curves have two properties consistent with identifying as that transition. They differ from normal distributions in having highly negative skewness (1.50 ± 0.4) and highly positive kurtosis (5.60 ± 2.6). The latter descriptor means that the distributions are much narrower than expected of normal distributions; the former means that they are highly asymmetric with larger tails at lower temperatures. That transition occurs very close to the growth temperature optimum of *G. stearothermophilus* (58–62 °C) and may, therefore, be functionally relevant.

### D. Multiple regression analysis demonstrates linearity of mutational impacts on stability

**FIG. 5.**
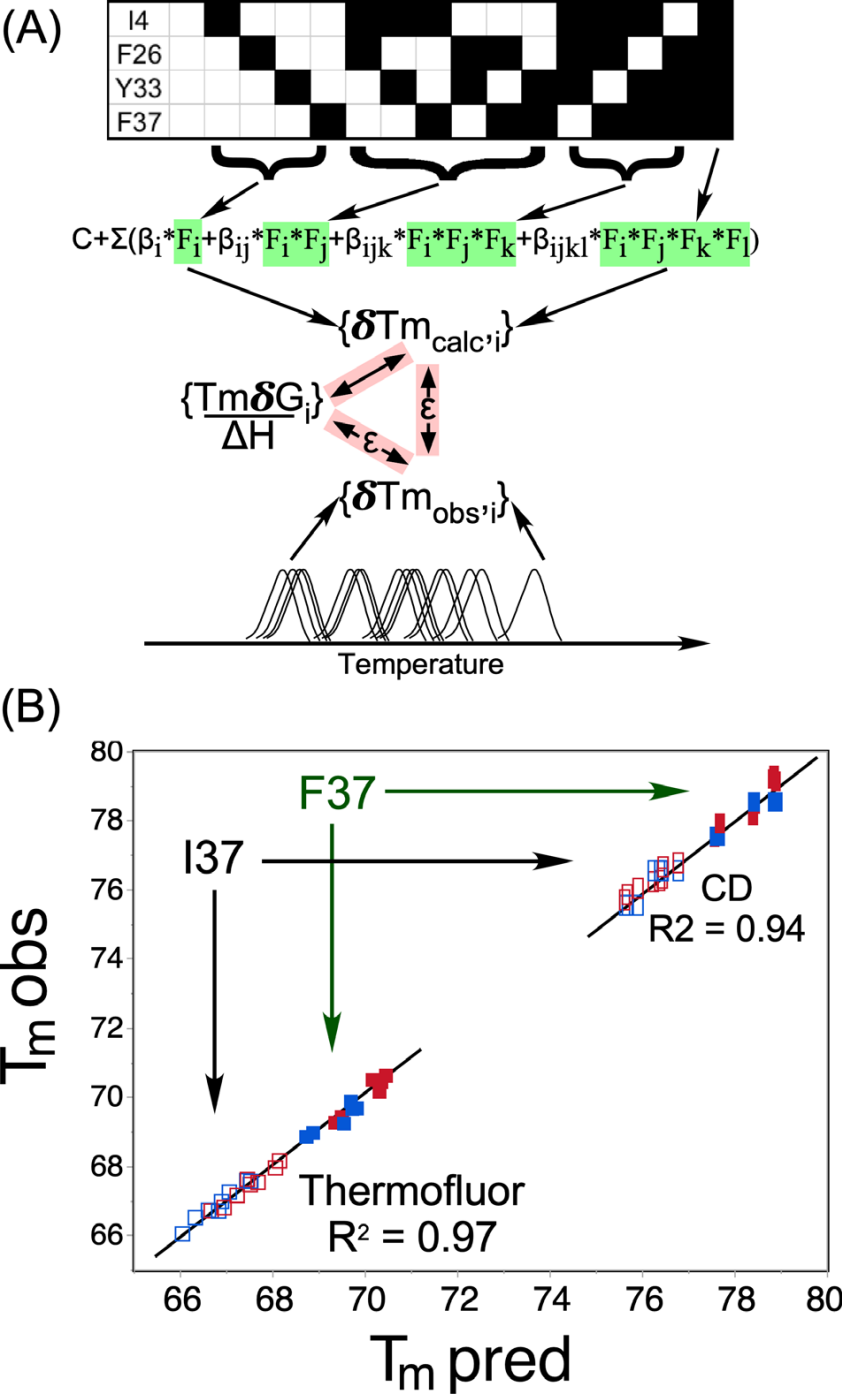
Linear self-consistency of T_m_ values calculated from regression models. (a) Thermodynamic cycle illustrating that the extent to which calculated and observed {δT_m,i_} values agree is a measure of how closely the measured values approach proportionality to incremental free energies {δG_i_}. The barcode represents the factorial design of combinatorial mutations and differentiates the four types of predictors in the simultaneous equations of the regression model. Observed T_m_ values for the variants are suggested schematically by the first derivatives of melting curves in Fig. 3. (b) Calculated T_m_ values for wild type and each of 15 combinatorial D1 switch mutants agree closely with observed values for both melting transitions, validating the regression models and the thermodynamic cycle in (a). Both ${{\;T}_{m}}_{\Phi }$ and ${T_{m}}_{\theta }$ values are clustered, according to whether residue 37 is F (solid) or I (empty). Although ${{\;T}_{m}}_{\Phi }$ and ${T_{m}}_{\theta }$ are associated with different β coefficients, Table I, as indicated by the break in the regression line, the two sets of modeled melting temperatures are nearly co-linear. Red symbols were measured in February, blue in May (2011), and were scaled by a single scaling factor, 0.62°, before evaluating coefficients.

The barcode in Fig. 5(a) represents the design matrix for the combinatorial mutagenesis, which represents a nearly ideal set of perturbations according to a balanced, factorial design. Because free energy is a state function, additivity of ${\delta T}_{m,\text{obs}}$ for the native and 15 variant proteins would imply proportionality to free energy perturbations via Eq. (1).^22^ The vector of 16 melting temperatures for each transition, thus, provides an implicit test of additivity.

The data constituting ${\{T}_{m\;\mathrm{obs}}\}$ is derived from replicated measurements from two different TrpRS purifications made three months apart and in the presence of 20 mM MgCl_2_. Regression models for both Thermofluor and CD melting temperatures are in Table I. Agreement between the two sets of data (i.e., the R^2^ values) is achieved using only a modest subset of the possible predictors. T_mφ_ and T_mθ_ values are linearly related by regression modeling to the matrix of mutated sites with 8 and 10 coefficients, corresponding to 8 and 6 degrees of freedom, respectively. Thus, both sets of T_m_ values for the ensemble of mutant proteins form very good approximations to legitimate thermodynamic cycles.

The schematic thermodynamic cycle in Fig. 5(a) shows that the smaller the difference vector, **ε** in (2), the better the approximation {δT_m calc,i_} is to {δT_m obs,i_}, and, consequently, the better the approximation {δT_m obs,i_} is to {δG_i_}. Figure 5(b) is, thus, evidence that conditions for (1) are satisfied for the entire ensemble of variants. Consequently, we can attribute both Thermofluor and CD δT_m,obs,i_ values to {δG_i_} values for both main and higher-order moments of the combinatorial design matrix.

The high R^2^ values, 0.97 and 0.98, respectively, of Thermofluor and CD multiple regression models together with Eq. (1) further imply that: (i) ΔH does not change significantly for different mutants for either transition; (ii) D1 mutations perturb stability largely by their different TδS terms; and (iii) ensembles of microstates that accumulate maximally at the transition temperatures are approximately at equilibrium, despite the observed aggregation (Sec. 1 of Ref. 53). The evident linearity and vectorial nature of the combinatorial mutagenesis, therefore, complement and strengthen the evidence from the computational free energy surface^19^ that TrpRS melting curves have meaningful thermodynamic interpretations.

### E. Wild type residue F37 stabilizes both native and molten globular states of unliganded TrpRS

**FIG. 6.**
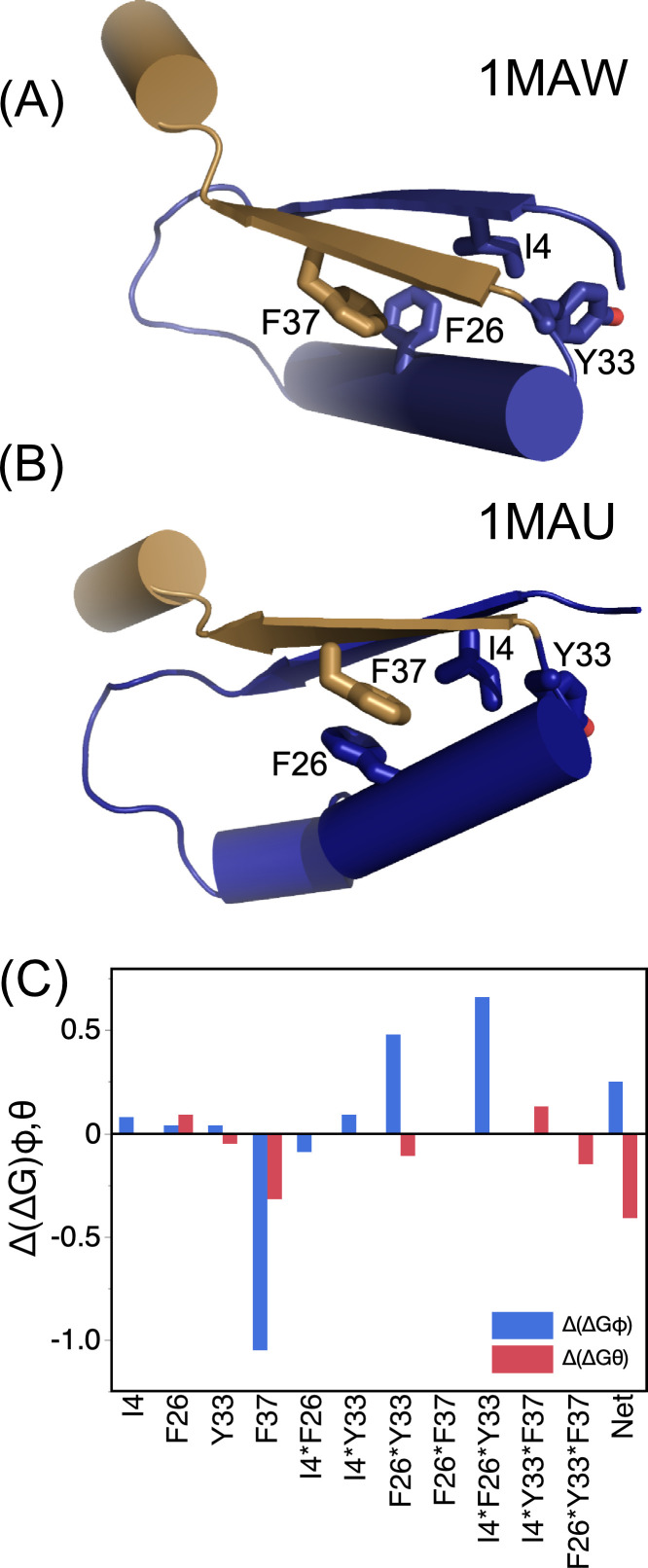
Wild type residue Phenylalanine 37 plays a predominant role in stabilizing unliganded TrpRS. The significance of the large β_φ_ and β_θ_ coefficients (c) can be attributed, in part, to its interactions within the first crossover connection in the Rossmann fold. (a) In the unliganded state, F37 packs onto F26 in approximately a “T” configuration, holding the second β-strand (sand) against the surface of the first crossover connection (dark blue). (b) In the PreTS state, that interaction shifts to a less stable,^52^ “parallel-displaced” orientation.

Mutational effects are both context- and reaction-dependent. Packing within the D1 switch changes as the active site assembles 20 Å away [Figs. 6(a) and 6(b)]. Combinatorial perturbations of the D1 switch residues begin disentangling how context dependence affects the inference of intrinsic conformational stabilization by its component residues from the melting behavior of point mutations themselves. In the process, we uncovered a second source of variation: TrpRS forms a separable molten globular melting intermediate; estimated contributions to stability, therefore, also depend on the denaturation stage. The stabilization energies [Fig. 6(c)] merit five comments:

i. The regression model for a 2^4^ factorial design^43^ has 16 independent parameters, including the constant term. Regression modeling, summarized in Table I, uses averaging to enhance the precision and sensitivity of mutational analysis, quantitation, and significance testing of high-order interactions (see supplement to Ref. 3). The 16 variants also balance comparisons of mutant behaviors. The intrinsic and net effects of each mutation on the stabilities of the unliganded native and molten globule states are averaged over eight, each double mutant over four, and each triple mutant over two different contexts [wild type and mutant in the remaining residue(s)].
ii. Curiously, and despite the pronounced stabilization by F37, higher-order interactions between D1 residues destabilize the molten globule transition. The net effect actually stabilizes α-helices relative to the molten globule [Fig. 6(c)]. Perhaps for that reason, estimates from point mutation measured by CD correlate with the intrinsic effects derived from regression analysis for molten globule formation (R^2^ = 0.95). Balanced measurements of context-dependence throughout the denaturation profile thus provide more reliable estimates of the intrinsic stability contributions of the four residues.
iii. Mutation induces temperature perturbations that agree closely with those computed from linear regression models based on the design matrix of mutation sites. This implies that they form a thermodynamic cycle with a relationship described by Calvin *et al.*^22^ relating perturbations in T_m_, δT_m_, to perturbations in folding free energy, δΔG.
iv. Wild type residue F37 from the D1 switch stabilizes both native and molten globular states. The D1 motif is a broadly conserved packing motif^14^ that creates the conformational transition state^15^ that precedes the catalytic step. Much of that free energy barrier arises because of the change in packing of F37, I4, and F26 [Figs. 6(a) and 6(b)]. Interactions between these residues yield a complex pattern of energies [Fig. 6(c)]. The net effect in unliganded TrpRS is to stabilize the open conformational state (1MAW), relative to the molten globule. Estimates in Table I imply that F37 stabilizes both native and molten globular states of unliganded TrpRS.
v. The linear dependence of δTm on the mutational design matrix (A) implies the feasibility of using Thermofluor also to link functionally relevant differential conformational stabilities across the reaction pathway to the design matrix. Measurements reported here represent only part of the data required to accomplish that goal.

All four stability estimates for the four D1 residues—i.e., from point mutants and main effects measured for both melting transitions—are correlated (R^2^ = 0.94, 0.94, 0.99, and 0.96) with the sums of the corresponding main effects from the two regression models (see Sec. 9 and Table SII of Ref. 53). That high correlation confirms the additivity of free energy estimates for the two melting transitions, further substantiating Eq. (1), hence our thermodynamic interpretations.

D1 residue packing appears likely to stabilize the marginal helix propensity of the urzyme's N-terminal helix. The net stabilizing effect of the D1 residues suggests that those forces arise from packing interactions within the D1 switch. Remarkably, the mean positions of the atoms in these four side chains remain within a root mean square distance of 2.5 Å of their relative positions in the 1MAW crystal structure of the open ATP complex. Wild type F37 stabilizes both native and molten globule states, elevating the temperature at which α-helices melt [Fig. 5(b)]. The effects of F37 are consistent with the fact that this residue binds the second β-strand of the Rossmann fold to the first α-helix, forming a dynamic tertiary interface [Figs. 6(a) and 6(b)]. Such stabilization probably helps ensure that the weaker N-terminal helix melts only when these tertiary interactions are broken as discussed further in Ref. 19.

Multi-mutant thermodynamic cycles yield structural insight into the molten globule state. Wild type F37 residue stabilizes both the unliganded open conformation against molten globule formation by ∼2.5° and the molten globule, relative to the denatured form by another ∼2.4°, almost irrespective of the variant in which it occurs [Fig. 5(b)]. Notwithstanding, the net cooperative impact of the D1 switch residues impacts Thermofluor and CD melting steps in opposite ways. Indeed, the net effect of the D1 switch residues destabilizes the molten globular state by ∼1 kcal/mole, despite the significant stabilizing effect of F37. That paradox arises from its interactions involving F26 and Y33, the other two aromatic residues [Fig. 6(c)].

## IV. DISCUSSION

Results described here highlight multiple ways in which high-throughput thermal melting analysis of combinatorial mutant ensembles can build on the protein landscape view of protein stability. First, they emphasize the utility of bioinformatic tools for identifying locally dynamic regions, thereby focusing attention on residues most likely to exhibit functionally relevant behavior. Second, they show that melting temperature changes measured for a full-factorial ensemble of variant proteins can yield legitimate free energy changes. Finally, we illustrate how bringing disparate kinds of structural analysis to bear can point forward to unexpected new questions. Together, these establish a paradigm for studying how locally dynamic interactions contribute functionality.

### A. Bioinformatic identification of locally dynamic packing

Protein landscapes are highly complex structures that pose substantial challenges to those interested in relating structure to function. Bioinformatic filtering via Delaunay Tessellation and likelihood scoring together with using protein-based physics in the design of combinatorial mutants furnishes a general paradigm for filtering the dynamic inter-residue coupling from the vast number of static packing configurations that contribute far less importantly to function.

Identification of the D1 packing motif^15^ initiated an extended series of experimental^1,3,5,6,16,18^ and computational^1,4,7,19,30^ studies relating mutant-induced structural changes quantitatively to both kinetic and computational parameters during catalysis. Results reported here extend the utility of being able to pinpoint structurally dynamic linkages.

### B. δT_M_ values in factorially designed combinatorial mutagenesis are proportional to thermodynamic free energies

The relative melting temperatures, δT_M_, contain legitimate comparative thermodynamic information:

(i) Relative widths, areas, and transition temperatures of Thermoflour and CD melting profiles match approximately to those obtained from Differential Scanning Calorimetric melting, as described in the accompanying paper,^19^ and which by definition is reversible.
(ii) All T_mφ_ and T_mθ_ values are linearly related by regression modeling to the matrix of mutated sites with 6 and 10 degrees of freedom and R^2^ = 0.97 and 0.95, respectively.

Regression methods are useful because they establish the linearity of the temperature perturbations, and hence their interpretation via the thermodynamic circle formed with Eq. (1). In that context, they have two additional non-trivial advantages:

(i) Ratios of model coefficients to their standard errors provide Student t-values, whose probabilities under the null hypothesis are known, giving statistical significance for each estimate and ensuring that models are not overfitted—i.e., that regression coefficients are estimated with sufficient degrees of freedom to estimate the noise.
(ii) The squared correlation coefficient between { $T_{m\;\mathrm{calc}}\}$ and { $T_{m\;\mathrm{obs}}\}$, R^2^, is equal to the fraction of the variance in $T_{m\;\mathrm{obs}}$ explained by the model. Moreover, high precision of the data collection is key to a conclusive interpretation of melting behavior because the maximum R^2^ that can expected for a given model, in turn, decreases linearly with the square of the relative error in the experimental data to which it is fitted.^44^ Data replication, therefore, enhances the signal-to-noise of regression models by reducing the relative error in the data points, even though for factorial experiments, it cannot increase the effective ratio of data to parameters. For this reason, Student t-test P-values are the key determinant of whether or not a model is overfitted.

Provided that models are not overfitted, R^2^ values close to 1.0 support causal relationships. This internal consistency of a regression model furnishes a metric for the extent to which the model may have omitted important causative variables. In our case, the relative size of the error vector, **ε**, the implied statistical significance of the Student t-tests, the absence of outliers, and the R^2^ value associated with regression models constitute an interrelated set of support that the melting temperatures have thermodynamic meaning, as discussed with reference to Fig. 5.

The high correlation coefficients illustrated in Fig. 5 underscore the far-reaching importance of Eq. (1)^22^ to high-resolution analysis of structure/function relationships. A dogmatic reluctance of the field to attribute thermodynamic significance to denaturation studies except in the rare circumstances that avoid aggregation and demonstrate high reversibility has long precluded the kinds of experiments documented here, in which moderately high-throughput methods such as Thermofluor facilitate estimation of incremental free energy changes induced by combinatorial mutagenesis.

The experimental enthalpy change for denaturation^19^ enables conversion of regression coefficients into thermodynamic free energy changes. Together with the efficient identification of dynamic side chain packing interactions described in Sec. IV A, that conversion opens the way to high-resolution mapping of a new class of dynamic linkages relevant to catalysis.

### C. Analysis of thermal melting directly implicates local frustration

An important novelty issuing from the protein landscape metaphor is the notion that side chain packing free energies vary along the polypeptide chain according to a local parameter called frustration.^45,47^ The algorithm compares pairwise interactions along the chain with a null hypothesis based on alternative packing arrangements with different residues and/or different environments.^45^ The sequence of TrpRS crystal structures affords a representative sample along the reaction coordinate for tryptophan activation, including a structure, 2OV4, complexed with the transition-state analog, adenosine′5′tetraphosphate.^46^

We previously commented on the fact that frustratograms derived from those crystal structures show that frustrations within the three regions of greatest local frustration all diminish significantly in the transition-state analog complex (see Fig. 4 of Ref. 48). We proposed therein that the reduced local frustration was akin to a local melting in the presence of a ligand resembling the chemical transition state. Our analogy at the time was that the mature TrpRS retained something of the flexibility we had attributed to the ability of the TrpRS urzyme to function as a catalytically active molten globule. Indeed, Hilvert had shown that a mutant chorismate mutase also characterized as a molten globule, formed a properly folded structure if and only if a transition analog was present.^49,50^

**FIG. 7.**
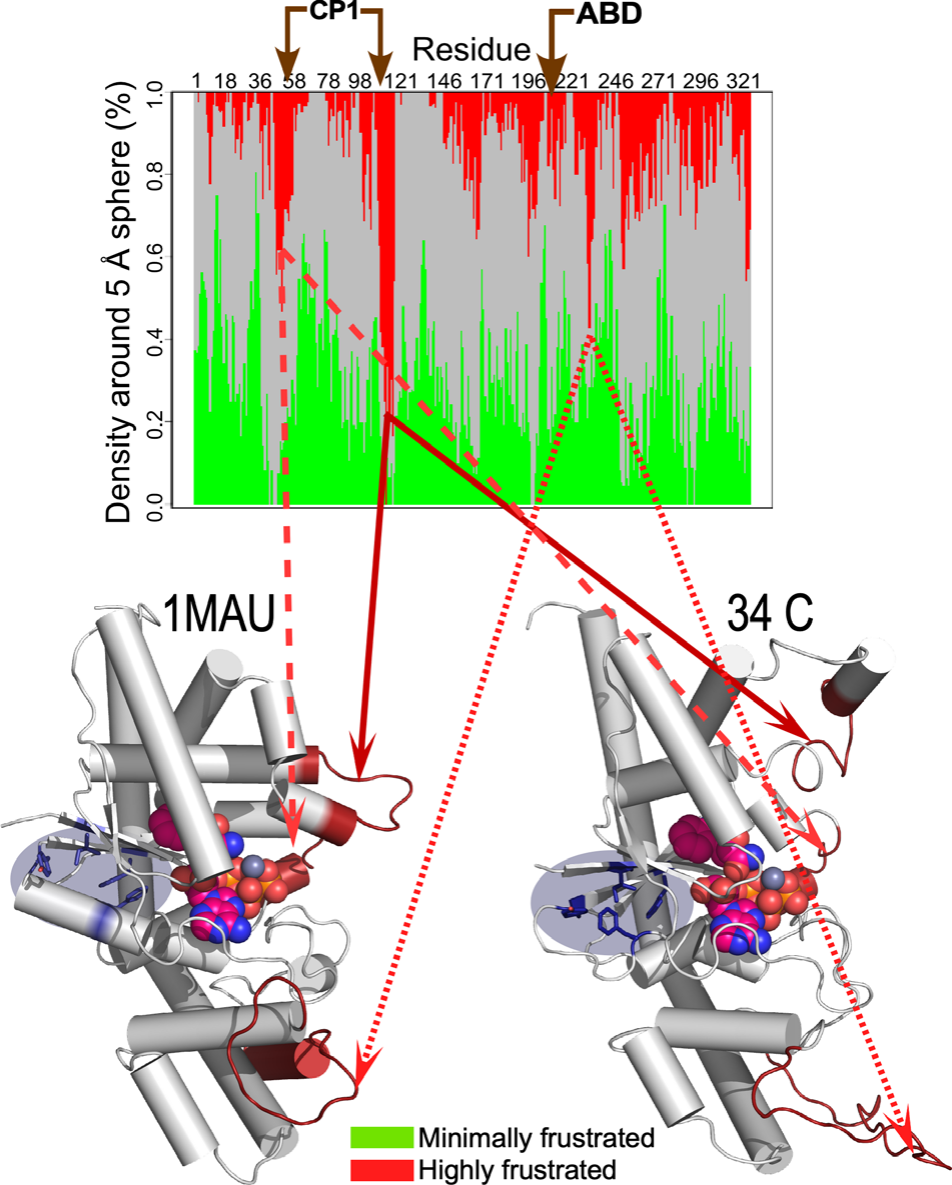
Correlations between regions of local frustration and early melting regions in TrpRS. Frustratogram derived from the 1MB2 crystal structure of the open tryptophan complex shows three localized regions of highly frustrated side chain packing (adapted from Ref. 48). Those regions are identified with corresponding structures of the 1MAU crystal structure of the PreTS TrpRS complex with tryptophanamide and ATP (left) and the ensemble average structure at 34 C drawn from the replica exchange temperature-dependent free energy surface trajectory described in the accompanying paper for the PreTS state. The only regions that unfold at 34 C are closely correlated with the segments of maximum local frustration (pairs of red dashed lines). Those segments also correspond closely to the junctional connections between the TrpRS urzyme, the CP1 insertion, and the ABD as noted in the text. Curiously, the melting of these three segments is also associated with repacking of the D1 switch residues on the other side of the TrpRS monomer, which are highlighted by gray ellipses.

Figure 7 extends the observation in light of the present work. As expected from the theory of local frustration, minimal frustration along the TrpRS chain is divided into four domains, two of which—residues 1–46 and 120–204—compose the TrpRS urzyme, which remains folded at the highest temperature of the replica exchange DMD (Discreet Molecular Dynamics) trajectory.^19^ The other two regions of minimal frustration are the CP1 and anticodon-binding domains. Short, highly frustrated segments interrupt these four regions. Curiously, those frustrated segments are the first parts of the TrpRS monomer to denature in the thermal replica exchange trajectory.

Moreover, the D1 switch residues, which form a relatively destabilizing packing arrangement involving parallel stacking of two phenylalanine resides in the high-energy PreTS state [Fig. 6(b)], relax in the 34 C structure to a configuration similar to that observed in the open ground-state structures. It seems, therefore, that the configuration of the D1 switch residues is coupled not only to the active-site metal and to the domain configuration of CP1 and the anticodon-binding domain (ABD), but also to these segments of high local frustration. That unanticipated coupling points directly to aspects of the TrpRS mechanistic enzymology that must be investigated by subsequently designed experiments.

## V. CONCLUSIONS

Differential scanning fluorimetry (Thermofluor) and far UV circular dichroism and combinatorial mutagenesis of *G. stearothermophilus* Tryptophanyl-tRNA synthetase compose a paradigm for high-resolution mapping enzyme function to sequence within the protein stability landscape across the structural reaction profile. Thermofluor melting curves confirm that TrpRS denatures via a molten globular intermediate. One D1 switch residue, F37, dominates the stabilization of the unliganded native conformation, relative to both the molten globular intermediate and the fully denatured states despite unfavorable coupling to other D1 switch residues. Higher-order interactions, not evident in single mutations, actually destabilize the molten globular state.

These measurements open a new window on the study of the conformational behavior underlying the TrpRS escapement mechanism. Together with the accompanying paper,^19^ this work argues that combinatorial mutagenesis and high-throughput thermal melting experiments represent a new paradigm for high-resolution mapping of function to sequence across the folding landscape.

## Data Availability

The data that support the findings of this study are available within the article and its supplementary material.
